# Annual direct and indirect costs attributable to nocturia in Germany, Sweden, and the UK

**DOI:** 10.1007/s10198-016-0826-x

**Published:** 2016-09-27

**Authors:** Diana Weidlich, Fredrik L. Andersson, Matthias Oelke, Marcus John Drake, Aino Fianu Jonasson, Julian F. Guest

**Affiliations:** 1 Catalyst Health Economics Consultants, 34b High Street, Northwood, Middlesex HA6 1BN UK; 2 Ferring Pharmaceuticals, Copenhagen, Denmark; 3 Center for Medical Technology Assessment (CMT), Linköping University, Linköping, Sweden; 4 Department of Urology, Hanover Medical School, Hannover, Germany; 5 Bristol Urological Institute, Bristol, UK; 6 Division of Obstetrics and Gynaecology, CLINTEC, Karolinska Institutet, Stockholm, Sweden; 7 Faculty of Life Sciences and Medicine, King’s College, London, UK

**Keywords:** Burden, Cost, Lower urinary tract symptoms, Nocturia, Germany, Sweden, UK

## Abstract

**Objective:**

Our aim was to estimate the prevalence-based cost of illness imposed by nocturia (≥2 nocturnal voids per night) in Germany, Sweden, and the UK in an average year.

**Methods:**

Information obtained from a systematic review of published literature and clinicians was used to construct an algorithm depicting the management of nocturia in these three countries. This enabled an estimation of (1) annual levels of healthcare resource use, (2) annual cost of healthcare resource use, and (3) annual societal cost arising from presenteeism and absenteeism attributable to nocturia in each country.

**Results:**

In an average year, there are an estimated 12.5, 1.2, and 8.6 million patients ≥20 years of age with nocturia in Germany, Sweden, and the UK, respectively. In an average year in each country, respectively, these patients were estimated to have 13.8, 1.4, and 10.0 million visits to a family practitioner or specialist, ~91,000, 9000, and 63,000 hospital admissions attributable to nocturia and 216,000, 19,000, and 130,000 subjects were estimated to incur a fracture resulting from nocturia. The annual direct cost of healthcare resource use attributable to managing nocturia was estimated to be approximately €2.32 billion in Germany, 5.11 billion kr (€0.54 billion) in Sweden, and £1.35 billion (€1.77 billion) in the UK. The annual indirect societal cost arising from both presenteeism and absenteeism was estimated to be approximately €20.76 billion in Germany and 19.65 billion kr (€2.10 billion) in Sweden. In addition, in the UK, the annual indirect cost due to absenteeism was an estimated £4.32 billion (€5.64 billion).

**Conclusions:**

Nocturia appears to impose a substantial socioeconomic burden in all three countries. Clinical and economic benefits could accrue from an increased awareness of the impact that nocturia imposes on patients, health services, and society as a whole.

## Introduction

Individuals who awaken one or more times at night to void, with each void being preceded and followed by sleep, are considered to have nocturia [1]. However, those who experience less than two voids per night are generally not regarded as having clinically significant nocturia that warrants diagnostic investigation and treatment. In contrast, people who experience two or more on a regular basis are perceived to be experiencing a significantly bothersome and clinically meaningful condition [2–4]. One consequence of persistent nocturia is associated sleep fragmentation, suggested by some to be more bothersome than nocturnal voiding frequency per se [5]. Sleep fragmentation affects the most restorative stage of sleep (slow-wave sleep), which could have potentially deleterious impact on daytime alertness, health, and well-being [6, 7].

While nocturia is usually defined as awakening two or more times at night to void [2, 3], nocturnal polyuria is defined as “abnormally excessive urination during the night (>33 % of urine production in 24 h in elderly individuals)” [8]. Pathophysiologically, nocturia is frequently attributed to nocturnal polyuria (nocturnal urine overproduction) [9], which is often due to an altered endogenous production of arginine vasopressin hormone. Nocturia can also be a consequence of a range of causes, such as congestive heart failure, cirrhosis of the liver, obstructive sleep apnea, nephrotic syndrome, chronic renal disease, advanced age, side-effects from drugs or low anatomical or functional bladder capacity (e.g., due to bladder-wall fibrosis, overactive bladder syndrome, or benign prostatic enlargement/bladder outlet obstruction) [1, 10].

The consequences of nocturia and associated poor sleep include daytime fatigue, reduced quality of life (QoL), mood disturbance, reduced productivity at work, poorer overall health, and increased falls and fractures [11]. Notwithstanding, nocturia is a relatively underreported condition, and the true extent of the problem in the population is difficult to estimate. Additionally, nocturia is often under- or misdiagnosed by the treating physician [12, 13]. These factors may contribute to the challenge of identifying patients for whom investigation and corresponding treatment would be indicated.

Prevalence estimates in the published literature vary and are affected by the population studied, the age range considered, and the definition of nocturia used. There have been previous attempts to estimate the costs associated with nocturia in Europe. However, they have generally focused on individual components of healthcare or indirect societal costs arising from lost productivity [14]. High-quality articles on the cost of illness associated with nocturia are generally lacking. Accordingly, the objectives of this study were to estimate the prevalence-based cost of illness imposed by nocturia (≥2 nocturnal voids per night) in Germany, Sweden, and the UK in an average year. Sweden was selected as it was considered to be representative of the Nordic countries. Germany and the UK were selected as they were considered to be representative of Western European countries. However, Germany’s healthcare system allows patients to contact specialists directly without prior referral from a family practitioner, whereas the UK’s healthcare system employs a primary-care gatekeeper system.

## Methods

### Study design

This was a retrospective modelling study based on a systematic review of published literature and information obtained from clinicians involved in managing nocturia in Germany, Sweden, and the UK.

### Literature review

A systematic literature review was performed by searching EMBASE, PubMed, and Centre for Reviews and Dissemination databases for relevant publications on nocturia (i.e., ≥2 nocturnal voids) published between January 2010 and June 2015. The search terms focused on epidemiology, clinical and health outcomes, management, resource use, costs, and productivity. After applying various related search terms in the databases, 2617 abstracts were obtained. Of these, 2368 studies were excluded because of duplication or lack of relevance. This generated 249 publications to review in full. A manual literature search was subsequently conducted using the citations in the papers. Published studies that reported epidemiological data for only one gender were excluded from the analysis.

Due to the scarcity of country-specific studies, some data from other European and non-European countries were also extracted and analyzed. However, the included countries’ healthcare systems were similar to Germany, Sweden, and the UK. Country-specific prevalence rates stratified by age group and gender were only available for Sweden [15].

Mean prevalence rates were calculated for Germany and the UK using data from Finland [16], Sweden [15], and the USA [17], as they were the only available studies reporting age- and gender-specific prevalence. No data were collected for people aged ≥65 years in Sweden [15] and ≥80 years in Finland [16]. Hence, the prevalence rates for these age groups, stratified by gender, were extrapolated by using the last three available data points from each study (Tables 1 and 2). An annual incidence of 0.102 (i.e., number of new cases per population at risk in a given 12-month period), annual remission rate of 0.167 (i.e., proportion of patients in remission), and mortality data [pooled Hazard ratio (HR) = 1.29] pertaining to nocturia were obtained from published meta-analyses [18, 19].

**Table 1:** Prevalence rates of nocturia in Sweden [15]

Age (years)	Male	Female
20–29	0.045	0.094
30–44	0.040	0.078
45–59	0.131	0.126
60–69*	0.214	0.313
70–79**	0.302	0.407
80–89**	0.389	0.525

* Data in Asplund (2005) [15] was only collected for people <65 years; it was assumed that the same rates were valid for people between 65 and 69 years

** Estimated as described in the text

**Table 2:** Estimated mean prevalence rates of nocturia used for Germany and the UK [15–17]

Age (years)	Male	Female
20–29	0.04	0.09
30–39	0.05	0.09
40–44	0.07	0.12
45–49	0.10	0.13
50–59	0.16	0.18
60–69	0.29	0.28
70–79	0.40	0.40
80–89	0.52	0.49

The incidence of the main serious adverse consequences of nocturia (i.e., falling and fracture) was generally reported for the older patient population. A weighted mean incidence for nocturia patients who fall during the night of 0.189 (i.e., proportion of patients who fall) was derived using data on patients aged ≥70 years [20, 21]. The distribution of fall-related fractures attributable to nocturia was applied to the incidence of fractures attributable to nocturia of 0.041 (i.e., proportion of patients with a fall-related fracture, both reported by Nakagawa et al. [22]) to estimate the annual number of patients who experience each fracture type in an average year. Based on these calculations, the incidence of fall-related accidents without subsequent fracture was estimated to be 0.148.

### Patient management

Due to the limited availability of published data, information was obtained from surveys investigating the management of nocturia patients in various countries. The number of nocturia-related consultations, hospitalizations, and over-the-counter drugs was obtained from a cross-sectional real-world survey involving 635 physicians who were actively managing urological patients in France, Germany, Spain, the UK, and the USA [23, 24]. Additionally, data on drug treatments were obtained from a nocturia market assessment survey in Europe [25]. Estimates collected from these surveys are summarized in Table 3.

**Table 3:** Information obtained from the surveys

Information obtained	Value	Survey
Mean number of nocturia-related consultations in a year: overall	3.6	[23]
Mean number of nocturia-related consultations in a year: family practitioner	4	[23]
Mean number of nocturia-related consultations in a year: urologist	3.4	[23]
Percentage of patients hospitalized because of nocturia in a year	2.6 %	[23]
Percentage of patients buying over-the-counter drugs in Germany	22 %	[23]
Percentage of patients seeking medical help	34 %	[25]
Percentage of patients seeing a urologist as a first point of contact	25 %	[25]
Percentage of patients seeing a family practitioner as a first point of contact	60 %	[25]
Percentage of patients seeing a gynecologist as a first point of contact	6 %	[25]
Percentage of patients seeing other specialists as a first point of contact	9 %	[25]
Percentage of patients ever consulted a urologist	47 %	[25]
Percentage of patients ever consulted a family practitioner	67 %	[25]
Percentage of patients ever consulted a gynecologist	11 %	[25]
Percentage of patients seen by a urologist or a gynecologist receiving prescribed drugs	53 %	[25]
Percentage of patients seen by a family practitioner receiving prescribed drugs	23 %	[25]
Percentage of patients receiving anticholinergics from a urologist	40 %	[25]
Percentage of patients receiving α-blockers from a urologist	40 %	[25]
Percentage of patients receiving 5α-reductase inhibitors from a urologist	15 %	[25]
Percentage of patients receiving antidiuretic hormone replacement from a urologist	10 %	[25]
Percentage of patients receiving anticholinergics from a family practitioner	50 %	[25]
Percentage of patients receiving α-blockers from a family practitioner	30 %	[25]
Percentage of patients receiving 5α-reductase inhibitors from a family practitioner	15 %	[25]
Percentage of patients receiving antidiuretic hormone replacement from a family practitioner	5 %	[25]
Percentage of patients receiving anticholinergics from a gynecologist	100 %	[25]
Percentage of patients receiving antidiuretic hormone replacement from a gynecologist	5 %	[25]
Percentage of patients referred to another specialist by a urologist or a gynecologist	13 %	[25]
Percentage of patients referred to another specialist by a family practitioner	36 %	[25]

The clinical authors involved in managing nocturia patients in Germany, Sweden, and the UK estimated that due to differences between healthcare systems in these three countries, patients can have diverse treatment pathways. In Sweden and the UK, patients would initially see a family practitioner who might refer them to a urologist, a gynecologist, or a urogynecologist for further assessment. However, in Germany, patients can seek medical help directly from any physician without first seeing a family practitioner.

The basic diagnostic tests for nocturia [e.g., frequency–volume chart, symptom-score questionnaire, urinalysis, urinary flow rate, measurement of postvoid residual (PVR) urine] are the same in all three countries. However, ultrasound tests are generally not used to evaluate the cause of nocturia in Sweden, and usually no additional fee specifically for this test would be charged in Germany. Therefore, the cost of diagnostic ultrasound tests were excluded from the base-case analysis in all three countries. The clinical authors considered that once patients received a prescribed drug, they would need to take it on an ongoing basis until their symptoms disappeared. If a drug ceased to be efficacious, patients would either need to switch to another drug or take combined medication.

Nocturia patients can also suffer from other conditions (e.g., diabetes, arterial hypertension, cardiac diseases, depression, urinary tract infections) that usually precede the onset of nocturia [26]. However, because of disturbed nocturnal sleep, they also suffer from fatigue, loss of attention and productivity during the day, falls, and bone fracture.

### Cost-of-illness models

**Fig. 1:**
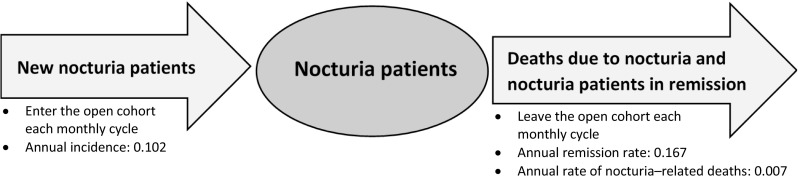
Cost-of-illness algorithm depicting the movement of nocturia patients entering and leaving the open cohort in an average year

Three country-specific cost-of-illness models comprising 12 one monthly cycles were constructed to estimate direct and indirect costs attributable to nocturia (≥2 nocturnal voids) in Germany, Sweden, and the UK in an average year (Fig. 1).

For each country, prevalence data (Tables 1 and 2) were used to estimate the annual number of patients aged 20–89 years who experience ≥2 nocturnal voids. Using published incidence rates, remission rates, and mortality HRs, our analysis estimated the annual number of newly diagnosed patients, patients who go into remission (i.e., experiencing <2 nocturnal voids) and discontinue treatment, and patients who die as a consequence of their nocturia (i.e., difference between the annual number of nocturia patients who die and the annual number of deaths in the general population using a mortality HR for nocturia [19]; background mortality for causes other than nocturia was not captured in the model). Hence, within the models, existing patients enter the model in the first month. New patients and patients in remission enter and leave the patient pool each month during the year. Additionally, deaths can occur in each month. The annual number of patients who enter or leave the pool each month in the three groups were distributed evenly throughout the year (by dividing annual incidence, remission, and mortality data by 12). Therefore, the size of the open cohort of patients at any one time depended on the number of newly diagnosed patients, patients who started treatment in a previous period, patients who died, and patients who went into remission. Annual amounts of healthcare resource use and corresponding costs were estimated separately for new patients and patients diagnosed with nocturia in previous years. However, the relative risk (RR) of having a fall or fracture was assumed to be the same for both newly diagnosed and existing patients.

In the base-case analysis, all patients from previous years were assumed to continue treatment for the year unless they went into remission or died. However, newly diagnosed patients were assumed to have to wait to see a specialist physician in accordance with country-specific average waiting times [27–29]. Moreover, patients generally received only lifestyle advice and a voiding diary to complete at the first visit. Accordingly, it was assumed that newly diagnosed patients would not start treatment until their second visit to a clinician, which would take place ~1 month after the first appointment. This impacts on the number of clinician visits and length of treatment experienced by newly diagnosed patients in the study year in the models. The number of falls and fractures and associated costs were estimated for both newly diagnosed and existing patients ≥70 years of age.

Indirect societal costs arising from absenteeism (i.e., being absent from work) and presenteeism (i.e., attending work when unwell, thus not working productively) were estimated for both newly diagnosed and existing patients aged between 20 and 64 years. Incremental values of absenteeism and presenteeism were estimated by calculating differences between nocturia patients [23, 30] and the general population [30–32] for each country. The costs of absenteeism and presenteeism were derived using national average monthly salaries [33] and the estimated incremental number of months that patients of working age are absent from work or do not work productively due to nocturia.

Due to a lack of data, presenteeism in the UK could not be estimated. Furthermore, based on findings from the available sources [23, 30, 31], people with nocturia in Germany and Sweden do not appear to be absent from work more than the general population. Therefore, it was assumed that there were no indirect costs arising from absenteeism due to nocturia in these two countries.

### Resource use and costs

Resource use was obtained from various sources: the two abovementioned surveys [23, 25], the clinical authors, and published literature [20–22]. Some data were not available; thus, the following assumptions were made:

- If a patient was referred to a specialist, only one additional specialist visit was assumed.
- Patients would initially visit their family practitioner in Sweden and the UK.
- Patients who have a fall would visit an accident and emergency department; those with fractures would require some type of fracture-related procedure in a hospital.

Unit costs at 2014–2015 prices were obtained from published sources (Table 4) and applied to the resource use estimates in the models to estimate the total annual direct cost of healthcare resource use and the annual indirect societal cost arising from presenteeism and absenteeism attributable to nocturia in the individual countries. If cost data was only available from previous years, values were uprated to 2014–2015 prices.

**Table 4:** Unit costs at 2014–2015 prices used in the base-case analysis

	Germany (€)	Sweden (kr)	UK (£)	Sources
Visits and tests				
Urologist	18.44	3886.00	99.00	UK [44], Germany [45], Sweden [46]
Family practitioner	16.51	1397.00	56.50	UK [47], Germany [45], Sweden [48]
Gynecologist	14.79	2489.00	135.00	UK [44], Germany [45], Sweden [46]
Other specialists	23.88	2933.00	133.00	UK [44], Germany [45], Sweden [46]
Diagnostic tests	0.00	854.00	1.00	UK [44], Sweden [49]
Drugs used per month				
Anticholinergics	38.49	251.12	23.09	UK [50], Germany [51], Sweden [52]
α-Blockers	20.66	130.91	5.59	UK [50], Germany [51], Sweden [52]
5α-Reductase inhibitors	23.56	82.42	15.42	UK [50], Germany [51], Sweden [52]
Antidiuretic hormone replacement therapy	121.50	345.02	27.32	UK [50], Germany [53], Sweden [52]
Over-the-counter drugs	5.40	0.00	0.00	[23]
Other related treatments				
Hospital bladder procedures	4982.97	35,424.94	1759.79	UK: [44], Germany [54, 55], Sweden [49]
Treatment after a fall	19.73	2323.50	135.00	UK [44], Germany [45], Sweden [49]
Hospital procedure to treat an arm fracture	4556.96	58,607.29	1939.83	UK [44], Germany [54, 55], Sweden [49]
Hospital procedure to treat a lower limb fracture	4037.71	66,273.80	1688.74	UK [44], Germany [54, 55], Sweden [49]
Hospital procedure to treat a lumbar spine and pelvic fracture	8409.20	63,380.60	2262.82	UK [44], Germany [54, 55], Sweden [49]
Hospital procedure to treat any other minor fracture	3898.36	56,448.03	1767.40	UK [44], Germany [54, 55], Sweden [49]
Indirect costs				
Average monthly gross salary	3042.83	31,468.08	2744.67	[33]

1 kr ≈ €0.11, £1 ≈ €1.31

### Sensitivity and scenario analyses

To assess whether any variable had a major impact on total direct or indirect costs of nocturia, one-way sensitivity analyses were performed on all model inputs. Base-case values were decreased and increased by 25 %. Various scenarios were also assessed to estimate the effect of increasing or decreasing the values of groups of variables (e.g., unit costs of drugs, number of visits in a year) and including the cost of an ultrasound test in the diagnostic tests. In addition, the impact of including the cost of treating depression, a condition that is suggested to have a bidirectional association with nocturia [34], was examined. In this scenario it was assumed that 10.82 % of patients had depression (a weighted mean estimated using Miyazato et al. (2014) [35] and Tikkinen et al. (2010) [36]) and needed corresponding treatment. Probabilistic sensitivity analysis was also undertaken to evaluate parameter uncertainty within the models. This involved 10,000 iterations of the models by simultaneously varying the different inputs. To estimate random values of inputs, the standard error (SE) was assumed to be 10 % around the mean values, and relevant distributions were assigned to the deterministic values. Beta distribution was used for probabilities, log-normal distribution for resource use estimates, and time variables and gamma distribution for costs enabling distributions of annual direct and indirect costs to be estimated.

## Results

### Prevalence of nocturia

Using prevalence data shown in Tables 1 and 2, the annual number of people aged ≥20 years experiencing two or more nocturnal voids per night was estimated (Table 5).

**Table 5:** Annual number of patients ≥20 years of age with nocturia in Germany, Sweden, and the UK

	Germany	Sweden	UK
Annual number of new patients	6,678,852	750,936	4,976,168
Annual number of existing patients	5,781,168	462,289	3,652,994
Total annual number of patients	12,460,020	1,213,225	8,629,162
Annual number of patients going into remission	2,080,823	202,609	1,441,070
Annual number deaths due to nocturia	93,399	8657	57,075

### Clinician visits

The estimated annual number of clinician visits attributable to managing patients experiencing ≥2 nocturnal voids per night is summarized in Table 6. Family practitioner visits account for more than half of all clinician visits in all three countries, and visits to a urologist account for another quarter.

### Prescriptions

An estimated 11 % of all sufferers in each country received prescribed drugs. Irrespective of country, 50 % of these patients received an anticholinergic, 32 % an α-blocker, 13 % a 5α-reductase inhibitor, and 8 % desmopressin (ADH replacement). Table 6 summarises the reimbursable drugs that are prescribed for nocturia patients.

**Table 6:** Annual amounts of healthcare resource use attributable to nocturia in Germany, Sweden, and the UK

	Germany	Sweden	UK
Clinician visits			
Annual number of visits to a family practitioner	7,722,803 (56 %)	811,774 (59 %)	5,826,666 (59 %)
Annual number of visits to a urologist	3,717,666 (27 %)	333,948 (24 %)	2,479,061 (25 %)
Annual number of visits to a gynecologist	934,985 (7 %)	84,298 (6 %)	624,562 (6 %)
Annual number of visits to other specialists	1,454,748 (11 %)	146,948 (11 %)	1,026,830 (10 %)
Total annual number of clinician visits	13,830,202 (100 %)	1,376,968 (100 %)	9,957,118 (100 %)
Prescribed drugs			
Total number of patients receiving prescribed drugs in a year*	1,404,931	133,707	963,033
Annual number of patients taking anticholinergics	705,371	66,957	482,904
Annual number of patients taking α-blockers	449,415	42,863	308,382
Annual number of patients taking 5α-reductase inhibitors	187,608	17,915	128,810
Annual number of patients taking desmopressin (ADH replacement)	107,346	10,202	73,533
Hospital activity			
Annual number of falls among patients aged ≥70 years	997,235	88,085	599,918
Annual number of fall-related fractures among patients aged ≥70 years	216,331	19,108	130,141
Annual number of hospital admissions	90,926	8857	63,038

Percentage of total number of visits in parentheses

*ADH* antidiuretic hormone

* Some patients can take a combination of drugs

### Falls and fractures

Twenty-two percent of falls among patients aged ≥70 years were estimated to result in a fracture. Of patients who had a fracture, it was estimated that 30 % had an arm fracture, 40 % had a lower limb fracture, 20 % had a lumber spine and pelvic fracture, and 10 % had a combination of other fracture types [22]. Additionally, 1 % of all patients were admitted into hospital (Table 6).

### Annual direct healthcare cost of managing nocturia

The direct annual cost of healthcare resource use attributable to managing nocturia was estimated to be approximately €2.32 billion in Germany, 5.11 billion kr (€0.54 billion) in Sweden, and £1.35 billion (€1.77 billion) in the UK (Table 7). Falls and fractures were the primary cost driver in Germany, accounting for 48 % of annual costs. In Sweden and the UK, clinician visits were the primary cost driver accounting for 59–60 % of the annual cost.

**Table 7:** Total annual direct healthcare cost (at 2014–2015 prices) of managing nocturia in Germany, Sweden, and the UK

	Total direct annual healthcare costs attributable to managing nocturia					
	Germany (€)		Sweden (kr)		UK (£)	
Clinician visits	244,625,051 (11 %)		3,072,586,752 (60 %)		795,517,872 (59 %)	
Prescribed drugs	472,403,515 (20 %)		194,126,227 (4 %)		137,443,084 (10 %)	
Over-the-counter drugs	37,607,278 (2 %)		0 (0 %)		0 (0 %)	
Diagnostic tests	0 (0 %)		180,073,118 (4 %)		1,398,160 (0 %)	
Hospitalization	453,083,943 (20 %)		313,770,214 (6 %)		110,934,067 (8 %)	
Falls and fractures	1,108,709,552 (48 %)		1,352,870,393 (26 %)		308,963,298 (23 %)	
Total direct cost	2,316,429,339 (100 %)		5,113,426,704 (100 %)		1,354,256,481 (100 %)	
Total direct cost per patient	185		4215		157	
Total direct cost per capita	29		525		21	

1 kr ≈ €0.11, £1 ≈ €1.31. Percentage of total cost in parentheses

### Annual indirect societal cost attributable to nocturia

In Germany and Sweden, it was estimated that patients of working age do not take time off work due to their nocturia [23, 30, 31]. Instead, they were estimated to not work productively for a total of ~1 month in an average year. In contrast, patients in the UK were estimated to be absent from work for a total of 0.4 months in an average year. However, no data were available to estimate their productivity at work (Table 8). Accordingly, the annual indirect societal cost arising from both presenteeism and absenteeism was estimated to be approximately €20.76 billion in Germany and 19.65 billion kr (€2.10 billion) in Sweden. In the UK, the annual indirect cost due to absenteeism was an estimated £4.32 billion (€5.64 billion) (Table 8).

**Table 8:** Absenteeism and presenteeism due to nocturia and corresponding total annual indirect societal cost due to lost productivity (at 2014–2015 prices) in Germany, Sweden, and the UK

	Germany	Sweden	UK
Percentage of patients of working age (i.e., 20–64 years)	49 %	48 %	51 %
Number of months not working productively due to nocturia (per patient)	1.13	1.06	N/A
Number of months absent from work due to nocturia (per patient)	0.00	0.00	0.36
Total annual indirect costs due to presenteeism	€20,759,295,433	19,647,399,994 kr	N/A
Total annual indirect costs due to absenteeism	€0	0 kr	£4,316,264,416
Total indirect cost	€20,759,295,433	19,647,399,994 kr	£4,316,264,416*
Total indirect cost per patient	€1666	16,194 kr	£500
Total indirect cost per capita	€256	2016 kr	£67

1 kr ≈ €0.11, £1 ≈ €1.31

* This only includes indirect costs due to absenteeism

### Sensitivity and scenario analyses

The one-way sensitivity analyses indicated two inputs that had the most significant impact on direct healthcare costs in all three countries. Changing (1) the annual number of nocturia patients and (2) the percentage of patients who seek medical help by ±25 % increased and decreased direct costs by 19–29 % and 13–19 %, respectively. In scenarios (Table 9) in which the number or unit cost of all types of visits were modified by ±25 %, direct healthcare costs increased and decreased most considerably in Sweden (by 15 % in both scenarios) and the UK (by 14–15 %) and much less in Germany (by 3 % in both scenarios).

**Table 9:** Total annual direct and indirect costs estimated with one-way sensitivity and scenario analyses in Germany, Sweden, and the UK

Scenario	Germany (billion euros)		Sweden (billion kronas)		UK (billion pounds)	
	Direct costs	Indirect costs	Direct costs	Indirect costs	Direct costs	Indirect costs*
Base-case result	€2.32	€20.76	5.11 kr	19.65 kr	£1.35	£4.32
Change the number of patients by ±25 %	€1.87–2.77	€15.57–25.95	3.75–6.51 kr	14.74–24.56 kr	£0.97–1.74	£3.24–5.40
Change the number of patients seeking medical help by ±25 %	€2.01–2.62	No effect	4.17–6.05 kr	No effect	£1.09–1.62	No effect
Change the annual number of clinician visits by ±25 %	€2.25–2.38	No effect	4.33–5.90 kr	No effect	£1.16–1.55	No effect
Change the percentage of patients receiving drug prescriptions by ±25 %	€2.20–2.43	No effect	5.06–5.16 kr	No effect	£1.32–1.39	No effect
Change the percentage of hospitalized patients by ±25 %	€2.20–2.43	No effect	5.03–5.19 kr	No effect	£1.33–1.38	No effect
Change the percentage of patients with a fracture by ±25 %	€2.04–2.59	No effect	4.82–5.41 kr	No effect	£1.29–1.42	No effect
Change the unit costs of clinician visits by ±25 %	€2.26–2.38	No effect	4.35–5.88 kr	No effect	£1.16–1.55	No effect
Change the costs of drugs by ±25 %	€2.19–2.44	No effect	5.06–5.16 kr	No effect	£1.32–1.39	No effect
Change the unit costs of hospitalization and nocturia-related procedures by ±25 %	€1.93–2.71	No effect	4.70–5.53 kr	No effect	£1.25–1.46	No effect
Include the cost of an ultrasound test as within the diagnostic tests	€2.35	No effect	5.74 kr	No effect	£1.43	No effect
0–2 months in a year of not working productively due to nocturia	No effect	€0–36.85	No effect	0–37.00 kr	N/A	N/A
0–1 month in a year of being absent from work due to nocturia	N/A	N/A	N/A	N/A	No effect	£0–12.15
40–60 % of patients are of working age (20–64 years)	No effect	€17.09–25.63	No effect	16.22–24.33 kr	No effect	£3.37–5.05

1 kr ≈ €0.11, £1 ≈ €1.31

* This only includes indirect costs due to absenteeism

Including the cost of an ultrasound test within the cost of diagnostic tests increased direct costs to a greater extent in Sweden (by 12 %) and to a smaller extent in Germany (by 1 %) and the UK (by 5 %). In the scenario in which the cost of depression was also taken into account, the cost per case in each of the three countries was obtained from a European-wide study [37] in which the cost per case included both direct and indirect costs of different brain disorders. Hence, using these estimates might have resulted in a double-counting of indirect costs. Consequently, the total annual direct and indirect cost attributable to nocturia would increase by approximately €11.51 billion in Germany, 6.49 billion kr (€0.69 billion) in Sweden, and £4.15 billion (€5.41 billion) in the UK if the cost of depression was included in the analysis. Probabilistic sensitivity analysis (10,000 iterations of the models) estimated mean total annual direct healthcare costs of:

- €2.24 billion [95 % confidence interval (CI) €2.21–2.28 billion) in Germany
- 5.10 billion kr (95 % CI 4.98–5.14 billion) [€0.54 billion (95 % CI 0.53–0.55)] in Sweden
- £1.34 billion (95 % CI 1.32–1.36 billion) [€1.75 billion (95 % CI 1.72–1.78)] in the UK.

Similarly, this analysis estimated mean total annual indirect societal costs of:

- €20.80 billion (95 % CI 20.46–21.13 billion) in Germany
- 19.50 billion kr (95 % CI 19.20–19.79 billion) [€2.08 billion (95 % CI 2.05–2.11)] in Sweden
- £4.33 billion (95 % CI 4.26–4.40 billion) [€5.65 billion (95 % CI 5.56–5.75)] in the UK.

## Discussion

This study estimated the economic burden that nocturia imposes on healthcare systems and society as a whole in three typically different European countries: Germany, Sweden, and the UK. The total annual direct healthcare cost of £1.4 billion attributable to managing nocturia in the UK is comparable with the annual National Health Service (NHS) cost of managing dementia (£1.5 billion at 2013–2014 prices) [38]. The total annual direct healthcare cost of €2.3 billion attributable to managing nocturia in Germany is comparable with the total annual direct healthcare cost of managing diseases of the thyroid gland and acute upper respiratory tract infections (€2.3 billion and €2.2 billion at 2014–2015 prices respectively) in Germany [39]. The total annual direct healthcare cost attributable to managing nocturia in Sweden (5.1 billion kr) is concordant with the total annual direct healthcare cost of managing overactive bladder syndrome (3.7 billion kr at 2014–2015 prices) in Sweden [40]. Comparable costs for managing overactive bladder syndrome in Germany and the UK were estimated to be €1.4 billion and £0.9 million, respectively [40].

The total annual direct healthcare cost of managing a nocturia patient in Germany (€186 per patient) and the UK [€205 (£157) per patient] were comparable. However, the corresponding cost in Sweden [€449 (4215 kr) per patient] was more than twofold greater that in the other two countries. The reason appears to be the unit cost of clinician visits and most hospital procedures in Sweden being higher than in the other two countries (Table 3).

We were unable to stratify our findings according to patient age. However, younger patients may experience more troubled sleep and associated problems than older patients [41]. Irrespective of whether sleep interruptions are caused or exacerbated by nocturia, these may affect the general state of health and well-being of sufferers. Furthermore, the negative effects of nocturia may be particularly difficult for younger patients because they are more likely to have more active lifestyles and demanding work schedules than older patients [42]. As proportionally more younger than older adults are expected to be employed, nocturia is seen as having a disruptive economic impact that is disproportional to the prevalence of this condition relative to that of older adults. Indeed, we estimated that the total annual indirect societal cost attributable to nocturia ranges from €20.8 billion in Germany to 19.7 billion kr in Sweden to £4.3 billion in the UK. Notwithstanding, others have reported that older patients with moderate to severe nocturia (≥3 nocturnal voids) incur significantly higher total medical costs, greater number of hospitalization days, higher inpatient medical costs, and higher outpatient medical costs than younger patients [43]. Furthermore, our analysis suggests there is no absenteeism from work as a direct result of nocturia in Germany and Sweden. By way of comparison, this would not appear to be the case for patients with overactive bladder syndrome in these countries [40]. Hence, the annual indirect societal cost attributable to overactive bladder syndrome has been estimated to be €441.3 million in Germany, 726.4 million kr (€77.38 million) in Sweden, and £199.0 million (€259.84 million) in the UK at 2014–2015 prices [40].

This analysis has several limitations: it is based on a systematic review of published literature and clinician experiences. Accordingly, the models were constructed by combining data from numerous, and in many instances non-European, countries with assumptions derived from the clinical authors. Additionally, patient-level data were not available for the study. As the clinical basis of the models was diverse studies, patient populations may not be identical in all of them. Consequently, the modelled patient populations may not necessarily reflect patients who are managed in clinical practice in Germany, Sweden, and the UK, and the observed clinical outcomes may not necessarily reflect those observed from patient cohorts in clinical practice.

The models were based on many assumptions pertaining to prevalence, incidence, mortality, and remission rates. The effect of all these limitations and the uncertainty surrounding the results underwent sensitivity analyses. Nevertheless, the results may be subject to unknown confounders.

The published literature describes various comorbidities that might be associated with nocturia. However, there is no clear evidence on the causal relationships between these comorbidities and nocturia [26]. Consequently, it was decided to exclude the cost of managing those comorbidities where there was any ambiguity regarding their relationship with nocturia. Nevertheless, the analysis can be updated once additional evidence becomes available. Notwithstanding, a scenario analysis estimated the impact of including the cost of depression, which was reported in a systematic review [34] to have a bidirectional association with nocturia.

Healthcare resource use was not collected prospectively but was estimated retrospectively from surveys and the clinical authors. Consequently, resource use for the “average clinician” may not be the same as for clinicians who participated in this study and may not be indicative of the entire populations of Germany, Sweden, or UK. The models incorporated resource use values for an “average patient” and did not take into account disease stage and patient characteristics such as age, gender, and comorbidities. In addition, the possibility of changing treatments (i.e., varying the number of different drug prescriptions) within a year was excluded from the analysis due to scarce applicable data; similarly, resource use and corresponding costs of managing patients in remission were excluded.

The study was performed from the perspective of the health service in each country and society as a whole. However, direct costs incurred by patients (including any co-payments), families, and/or caregivers were excluded (except for over-the-counter medication in Germany) due to the paucity of data. Inclusion of all missing costs (e.g., patient co-payments, cost of managing patients in remission) may affect study results and warrant further investigation. Therefore, generalizing results of this study to other healthcare systems would be challenging. Although healthcare systems in these three countries differ from each other, they could be viewed as being representative of similar types of healthcare structures. Hence, study results for Germany could be generalized to other Western European countries where patients can visit a specialist without a prior referral from a family practitioner. Conversely, study results for the UK could be indicative of other Western European countries with a primary-care gatekeeper system. Furthermore, results for Sweden could be representative of other Nordic countries.

In conclusion, nocturia appears to impose a substantial socioeconomic burden in Germany, Sweden, and the UK. Clinical and economic benefits could accrue from an increased awareness of the impact that nocturia imposes on patients, health services, and society as a whole.

## References

[1] van Kerrebroeck P (2002). The standardisation of terminology in nocturia: report from the standardisation sub-committee of the International Continence Society. Neurourol. Urodyn..

[2] Coyne KS (2003). The prevalence of nocturia and its effect on health-related quality of life and sleep in a community sample in the USA. BJU Int..

[3] van Dijk L, Kooij DG, Schellevis FG (2002). Nocturia in the Dutch adult population. BJU Int..

[4] Oelke M, Wiese B, Berges R (2014). Nocturia and its impact on health-related quality of life and health care seeking behaviour in German community-dwelling men aged 50 years or older. World J. Urol..

[5] van Dijk L (2004). Nocturia: impact on quality of life in a Dutch adult population. BJU Int..

[6] Bliwise DL, Dijk D-J, Juul KV (2015). Nocturia is associated with loss of deep sleep independently from sleep apnea. Neurourol. Urodyn..

[7] Holm-Larsen T (2013). “My sleep pattern is a series of naps”. Subjective patient-reported data about what is most bothersome about nocturia. Eur. Urol. Suppl..

[8] Abrams P (2002). The standardisation of terminology of lower urinary tract function: report from the standardisation sub-committee of the International Continence Society. Neurourol. Urodyn..

[9] Weiss JP (2011). Excessive nocturnal urine production is a major contributing factor to the etiology of nocturia. J. Urol..

[10] Weiss JP (2011). The evaluation and treatment of nocturia: a consensus statement. BJU Int..

[11] Asplund R (2005). Nocturia: consequences for sleep and daytime activities and associated risks. Eur. Urol. Suppl..

[12] Goessaert AS (2014). Extent to which a voiding diary is used to reach diagnosis in nocturia patients: results of a real world survey of physicians and patients in Europe and the USA. Eur. Urol. Suppl..

[13] Weiss JP, Andersson FL, Juul VK (2016). Diagnosing nocturnal polyuria (NP) based on self-reported nocturnal void volume and fluid intake in clinical practice: results from a real-world treatment survey in Europe and the USA. Eur. Urol. Suppl..

[14] van Kerrebroeck P, Holm-Larsen T (2011). The cost of nocturia in Europe. Int. Urogynecol. J..

[15] Asplund R (2005). Nocturia in relation to somatic health, mental health and pain in adult men and women. BJU Int..

[16] Tikkinen KAO (2006). Is nocturia equally common among men and women? A population based study in Finland. J. Urol..

[17] Kupelian V (2011). Association of nocturia and mortality: results from the third national health and nutrition examination survey. J. Urol..

[18] Pesonen JS (2014). Incidence and remission of nocturia: a systematic review of longitudinal population-based studies with meta-analysis and meta-regression. Eur. Urol. Suppl..

[19] Pesonen JS (2014). The impact of nocturia on mortality: a systematic review and meta-analysis. Neurourol. Urodyn..

[20] Stewart RB (1992). Nocturia: a risk factor for falls in the elderly. J. Am. Geriatr. Soc..

[21] Jamsen, E., Jantti,P., Nuotio, M.: Nocturia in hip fracture patients., in European Geriatric Medicine. 6th Congress of the EUGMS Dublin. Dublin. pp. S4–S5 (2010)

[22] Nakagawa H (2008). Does nocturia increase fall-related fractures and mortality in a community-dwelling elderly population aged 70 years and over? Results of a 3-year prospective cohort study in Japan. Neurourol. Urodyn..

[23] Adelphi Real World, LUTS Disease Specific Programme. Report prepared for Ferring (2013). Bollington.

[24] Anderson P (2008). Real-world physician and patient behaviour across countries: disease-specific programmes—a means to understand. Curr. Med. Res. Opin..

[25] IMS, Nocturia market assessment—primary market research. IMS Market Research Report prepared for Ferring Pharmaceuticals, October 2012 (2012)

[26] Yoshimura K (2012). Correlates for nocturia: a review of epidemiological studies. Int. J. Urol..

[27] HSCIC. Hospital Episode Statistics for England. Outpatient statistics, 2013–14. http://www.hscic.gov.uk/catalogue/PUB16722 (2015). Accessed 11 Oct 2015

[28] Roll, K., Stargardt, T., Schreyögg,J.: Effect of type of insurance and income on waiting time for outpatient care. Hamburg Center for Health Economics (2011)

[29] Anell A, Glenngård AH, Merkur S (2012). Sweden: health system review. Health Syst. Transit..

[30] Kobelt G, Borgstrom F, Mattiasson A (2003). Productivity, vitality and utility in a group of healthy professionally active individuals with nocturia. BJU Int..

[31] Bödeker, W., Hüsing,T., IGA-REPORT 12: IGA-Barometer 2. Welle. BKK Bundesverband (2012)

[32] ONS, Full Report: Sickness Absence in the Labour Market, February 2014. Office for National Statistics (2014)

[33] OECD. Average annual wages. http://stats.oecd.org/ (2014). Accessed 6 Oct 2015

[34] Breyer BN (2013). The association of depression, anxiety and nocturia: a systematic review. J. Urol..

[35] Miyazato M (2014). Prevalence and risk factors for nocturia in an outpatient clinic. Low Urin. Tract Symptoms.

[36] Tikkinen KA (2010). Nocturia frequency, bother, and quality of life: how often is too often? A population-based study in Finland. Eur. Urol..

[37] Andlin-Sobocki P (2005). Cost of disorders of the brain in Europe. Eur. J. Neurol..

[38] Knapp M (2007). Dementia UK: a report into the prevalence and cost of dementia.

[39] Statistisches Bundesamt: Krankheitskostenrechnung. http://www.gbe-bund.de (2008). Accessed 15 Jan 2016

[40] Irwin DE (2009). The economic impact of overactive bladder syndrome in six Western countries. BJU Int..

[41] Asplund R, Aberg H (1996). Nocturnal micturition, sleep and well-being in women of ages 40–64 years. Maturitas.

[42] Weiss JP (2007). Age related pathogenesis of nocturia in patients with overactive bladder. J Urol..

[43] Nakagawa H (2009). Impact of nocturia on medical care use and its costs in an elderly population: 30 month prospective observation of national health insurance beneficiaries in Japan. Neurourol. Urodyn..

[44] Department of Health. NHS reference costs 2013 to 2014. https://www.gov.uk/government/publications/nhs-reference-costs-2013-to-2014 (2014). Accessed 28 Sept 2015

[45] KBV. Einheitlicher Bewertungsmaßstab (EBM): Stand: 1. Quartal 2014. http://www.kbv.de/media/sp/Einheitlicher_Bewertungsma_stab_Stand_2014_1._Quartal.pdf (2014). Accessed 30 Sept 2015

[46] Socialstyrelsen and SKL. VÅRDKOSTNADER 2013 för NordDRG. http://skl.se/download/18.27a2758c14b3091437e9893f/1424103634046/V%C3%A5rdkostnader+NordDRG+2013.pdf#search='drg+pris' (2013). Accessed 30 Sept 2015

[47] PSSRU. Unit Costs of Health and Social Care 2014. http://www.pssru.ac.uk/project-pages/unit-costs/2014/ (2014). Accessed 29 Sept 2015

[48] SKL. Statistik om hälso- och sjukvård samt regional utveckling 2014. http://webbutik.skl.se/sv/artiklar/statistik-om-halso-och-sjukvard-samt-regional-utveckling-2014.html (2014). 30 Sept 2105

[49] Södra Regionvårdsnämnden. Regionala priser och ersattningar for sodra sjukvardsregionen. http://www.skane.se/Upload/Webbplatser/Sodra%20regionvardsnamnden/prislista/2015/helaprislistan2015.pdf (2015). Accessed 1 Oct 2015

[50] BNF. https://www.medicinescomplete.com/mc/bnf/current/ (2015). Accessed 1 Oct 2015

[51] DIMDI. https://portal.dimdi.de/festbetragsrecherche/ (2015). Accessed 5 Oct 2015

[52] TLV. http://www.tlv.se/beslut/sok/lakemedel/(2015).Accessed 05 Oct 2015

[53] Lauer-Taxe. www.lauer-fischer.de (2015). Accessed 15 Oct 2015

[54] InEK. http://www.g-drg.de/cms/G-DRG-System_2015/Fallpauschalen-Katalog/Fallpauschalen-Katalog_2015 (2015). Accessed 5 Oct 2015

[55] GKV-Spitzenverband. https://www.gkv-spitzenverband.de/krankenversicherung/krankenhaeuser/budgetverhandlungen/bundesbasisfallwert/bundesbasisfallwert.jsp#lightbox (2015). Accessed 7 Oct 2015

